# An analysis of hospital-treated attempted hanging and drowning in Ireland, 2007-2019

**DOI:** 10.1093/eurpub/ckac130.183

**Published:** 2022-10-25

**Authors:** P White, P Corcoran, E Griffin, E Arensman, P Barrett

**Affiliations:** 1 Department of Public Health, HSE-South, Cork, Ireland; 2 National Suicide Research Foundation, Cork, Ireland

## Abstract

**Background:**

Highly lethal of methods of self-harm, such as attempted hanging and drowning, are a major public health concern due to their high associated risk of completed suicide. This study aims to describe hospital presentations for attempted hanging and drowning in Ireland and explore the factors associated with self-harm and repeat self-harm by these methods.

**Methods:**

Data on all self-harm presentations to Irish hospitals (2007-2019) were obtained from the National Self-Harm Registry Ireland, a national surveillance system of hospital-treated self-harm. Multivariable logistic regression was used to explore factors associated with any presentation for attempted hanging and drowning and factors associated with repetition of attempted hanging and drowning.

**Results:**

There were 9,719 and 4,637 attempted hanging and drowning hospital presentations, respectively, in Ireland in 2007-2019. The odds of presentations being due to hanging, rather than due to any other self-harm method, were highest for males (aOR 2.88, 95% CI: 2.76-3.02), children aged <15 (aOR 1.32, 1.17-1.48) and in summer (aOR 1.09, 1.02-1.14). The odds of presentations being due to drowning, rather than due to any other self-harm method, were highest for those aged ≥55 (aOR 1.60, 1.43-1.78), homeless individuals (aOR 2.59, 2.32-2.89) and in autumn (aOR 1.15, 1.06-1.25). Repetition of attempted hanging was positively associated with homelessness (aOR 2.47, 2.02-3.04) and acute alcohol ingestion (aOR 1.12, 1.02-1.23). Similar associations were observed for repetition of attempted drowning.

**Conclusions:**

This study identifies key population groups for whom the risk of self-harm, or repeat self-harm, by hanging and drowning is greatest. Universal, targeted and indicated interventions are needed to address the determinants of highly lethal methods of self-harm. Biopsychosocial assessments of those presenting after attempted hanging and drowning are essential, in view of their high risk of repeat self-harm and suicide.

**Key messages:**

- Compared with other self-harm methods, the risk of self-harm by hanging and drowning may be highest among males and children aged <15 years and adults aged ≥55 and homeless individuals, respectively.
- A combination of interventions are needed to address self-harm by highly lethal methods.

